# A Comparative Analysis of Forming Railway Axles in 3- and 4-Roll Rolling Mills

**DOI:** 10.3390/ma13143084

**Published:** 2020-07-10

**Authors:** Zbigniew Pater

**Affiliations:** Department of Computer Modelling and Metal Forming Technologies, Faculty of Mechanical Engineering, Lublin University of Technology, Nadbystrzycka 36, 20-618 Lublin, Poland; z.pater@pollub.pl; Tel.: +48-81-538-4242

**Keywords:** railway axle, skew rolling, CNC rolling mill, FEM, experiment

## Abstract

The paper presents a comparative analysis of skew rolling in 3- and 4-roll CNC rolling mills. The analysis is performed using the FEM-based commercial simulation software Simufact.Forming. The formation of both solid and hollow railway axles is analyzed. Distributions of effective strain, temperature and damage criterion in rolled axles are determined, and loads and torques acting on the tools during rolling are estimated. An innovative concept of calibrating hollow axles by the rotary compression technique developed at the Lublin University of Technology is presented. Experimental tests of rolling solid axles in a 3-roll rolling mill (in a scale of 1:5) are performed, and basic force parameters of the rolling process are measured. Numerical and experimental loads and torques show a high agreement in terms of both quality and quantity.

## 1. Introduction

Railway axles are manufactured from large-size forged parts that are produced in batches of several thousand pieces [1,2,3]. Currently such axles are manufactured by open die forging and rotary swaging. Open die forging is performed in hydraulic presses (with a load ranging 8–15 MN, slide travel accuracy of ±1 mm and maximum speed of up to 90 strokes per minute) coupled with two manipulators (with an accuracy of 2 mm and ±1° for axial and angular positioning, respectively). Forgings of railway axles are formed from ingots with a circular or square cross section within a dozen minutes. The use of swaging machines reduces the forming time to 2–4 min. At the same time, the machines are provided with a system of 4 forming tools (each having a load of 5.6 MN), which does not allow good deformation of material in the core (axial area) of a forged part.

To increase the manufacturing efficiency and decrease energy consumption of the production process for railway axles, studies were undertaken to investigate the possibility of manufacturing these parts by rolling methods. Cross-wedge rolling (CWR), which is widely used for manufacturing stepped axles and shafts for the automotive industry [4,5], is a promising technique in this respect. What poses problems, however, is the axle size, as it directly affects the dimensions of machines and tools. The currently biggest 2-roll rolling mills of D46-1500 type have 1500 mm diameter rolls and make it possible to produce Ø 160 mm × 1300 mm parts. In turn, the biggest flat-wedge rolling mills, WRL20035TS, have 3.5 m long tools and make it possible to roll parts of up to Ø 200 mm × 1200 mm in size. In effect, the CWR machines are not suitable for producing railway axles, as such axles are considerably more than 2 m long.

The production of railway axles by the classical CWR method requires the use of approximately 6 m long tools. For this reason, studies were undertaken to investigate the possibility of forming these parts by multi-wedge rolling in which the workpiece is rolled by several pairs of wedges at the same time. Results of these studies [6,7,8,9,10] demonstrated that this rolling technique makes it possible to produce axles with a nominal diameter of approximately 1600 mm. It was also observed that the multi-wedge rolling process is more difficult to conduct due to difficulties related to removing cross sectional ovalization, especially when rolling hollow axles. In effect, a two-stage rolling process for railway axles was developed [11], in which the central step is formed first, and then end steps are rolled by the other pair of rolls. This solution allows reducing the diameter of the rolls to 1200 mm, which also causes a significant decrease in torque. For hollow axles, an innovative technique of rolling with three joint rolls was proposed [12], which ensures a rapid removal of cross sectional ovalization. It is also worth noting that in the aforementioned studies, numerical modelling was widely employed or rolling experiments were performed under laboratory conditions (usually, in a scale of 1:5).

The main disadvantage of cross-wedge rolling processes is the high cost of tools and the fact that they can be used for forming only one type of axle. Such limitations do not occur in 3-roll skew rolling in a CNC rolling mill. In this process, the workpiece is deformed by the rolls rotating in the same direction, as well as by the axially travelling chuck wherein the workpiece is mounted by one end. Apart from rotating, the rolls can move in a radial direction (toward the axis of the workpiece), which allows for changing the diameter of a rolled part.

Due to a lack of rolling stands, the first tests of skew rolling were only based on numerical simulations. The simulations showed that the method can be used for producing both solid and hollow railway axles [13,14,15]. In [16], it was shown that skew rolling requires relatively low loads and torques. Moreover, a great advantage of this process is the possibility of producing parts of different shapes using the same rolls [17,18]. These days rolling test stands are constructed that allow researchers to verify theoretical solutions. One of such rolling mills is available at the Lublin University of Technology and was used for the study described in this paper.

The proposed skew-rolling process performed in a CNC rolling mill is a modification of 3-roll skew rolling with a fixed distance between the axes of the rolls and workpiece. This process has been used for manufacturing seamless tubes and circular rods [19,20,21,22] for many years. In [23] Romantsev et al. demonstrated that the use of a 4-roll rolling mill for tube piercing leads to a significant reduction in energy required in the forming process. For this reason, an analysis was carried out to investigate the possibility of using the above rolling process for producing railway axles. Results of this analysis and their comparison with those obtained from the previously investigated skew-rolling process in a 3-roll CNC rolling mill are presented further in this paper. Summing up, the primary objective of this study is to investigate whether the use of 4 rolls for skew rolling railway axles is justified in terms of energy consumption of the forming process.

## 2. Scope of Analysis

**Figure 1:**
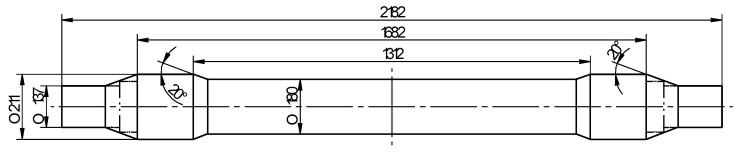
Analyzed railway axle (in a rolled form).

The object of the analysis was a BA3002-type railway axle (according to the EN1326 standard) shown in Figure 1. This axle is symmetric and modified in such a way that it can be formed in a CNC skew-rolling mill. The major modification is that the quasi-end step is substituted with a cone with an inclination angle of 20°.

**Figure 2:**
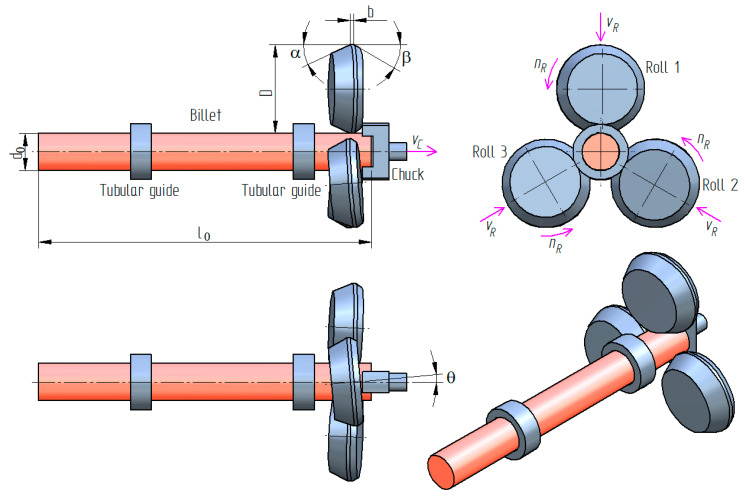
Schematic design of skew rolling in a 3-roll CNC rolling mill.

Figure 2 shows the scheme of the analyzed 3-roll skew-rolling process and its key parameters. The process of rolling with 4 rolls differs from that shown in Figure 2 with respect to the location of the rolls—they are located on the circumference of the workpiece at an angle of 90° in the former and at an angle of 120° in the latter.

**Figure 3:**
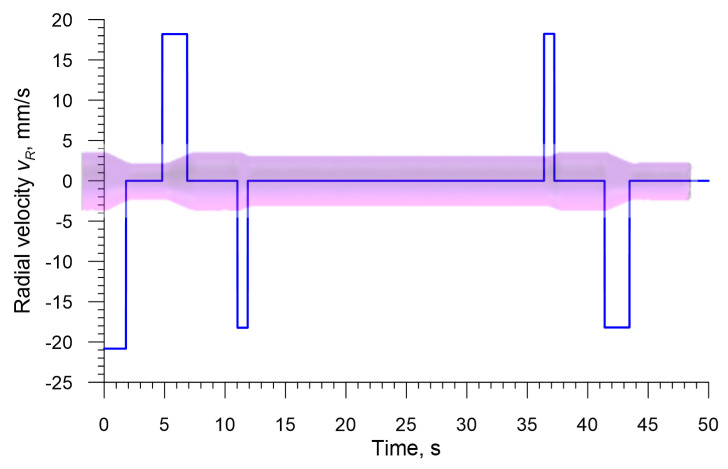
Radial velocity of the rolls, *v_R_*, used in the numerical analysis.

The rolls used in the two analyzed processes have different outside diameters: *D* = 500 mm (3-roll method) and *D* = 300 mm (4-roll method). Other parameters of the rolls are identical in both processes, i.e., *α* = 25°, *β* = 30° and *b* = 20 mm. The rolls are set askew to the axis of the workpiece at *θ* = 5°. The correct shape of a rolled axle depends on coupling the radial movement of rolls with the axial movement of the chuck. In the processes under analysis, the chuck moves with a constant axial velocity of *v_C_* = 50 mm/s during the entire forming process, whereas the radial velocity of the rolls, *v_R_*, changes during the rolling process, as shown in Figure 3. It is also worth noting that the rolls are rotated in the same direction at the constant rate of rotation *n_R_* = 60 rpm.

Four cases of rolling were analyzed. For each method two cases of rolling a solid and a hollow part were investigated. The solid axle was formed from a cylindrical rod of 211 mm in diameter and 1880 mm in length, whereas the hollow axle was formed from a tube with an outside diameter of 211 mm, a length of 2010 mm and a wall thickness of 40 mm. The dimensions of the billets included allowance for both chucking and end-face cavity that is formed when the final step is rolled on the solid axle. Moreover, the calibration of a hollow axle was analyzed; this process is necessary for forming steps on the axle ends.

Numerical simulations were performed using the commercial simulation program Simufact.Forming. This program had been effectively used before for analyzing both cross and skew-rolling processes [11,12,14,24,25,26]. The numerical results obtained showed a high level of agreement with experimental findings.

**Figure 4:**
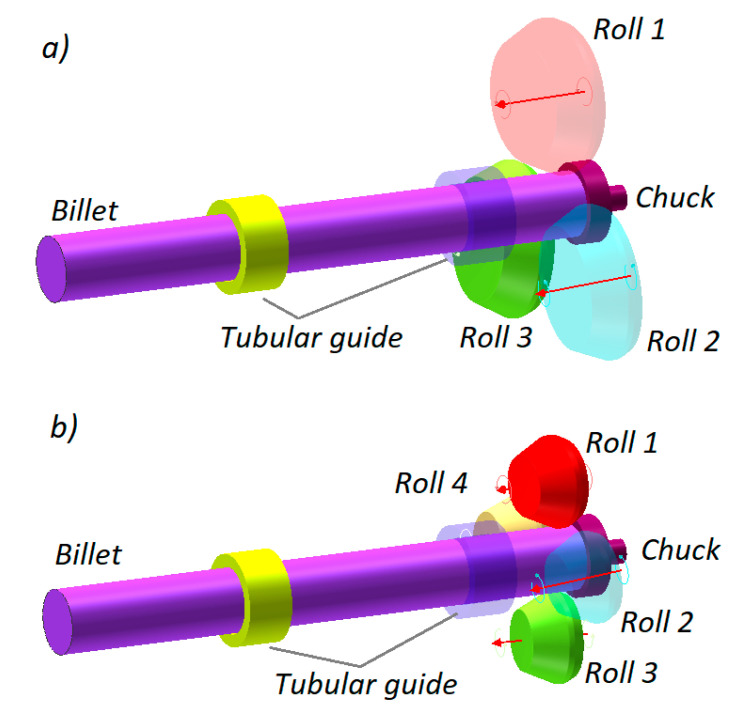
Geometrical models of rolling a railway axle from a solid billet: (**a**) in a 3-roll mill, (**b**) in a 4-roll mill.

Figure 4 shows two geometrical models of the analyzed rolling processes. Each model consists of rigid tools (rolls, chuck and tubular guides) and a billet that was modelled using 8-node hexahedral elements. The number of the elements was increased during the calculations due to, primarily, mesh refining resulting from a change in the workpiece shape, ultimately yielding a total of 41,000 elements. During the entire forming process that is 50 s long, the tools move in the above described manner. Additionally, the chuck can perform passive rotation caused by the rotating workpiece.

The model of the material used in the numerical analysis was the 42CrMo4 grade of steel (obtained from the material library database of the program), expressed by the following equation:

(1) $$\sigma_{F}=4628.8e^{-0.00345T}\varepsilon^{\left(-0.00000509T-0.03638\right)}e^{\left(-0.00000461T-0.01944\right)/\varepsilon }{\dot{\varepsilon }}^{\left(0.0001893T-0.04627\right)},$$

where *σ_F_* is the flow stress, *ε* is the effective strain, $\dot{\varepsilon }$ is the strain rate, *T* is the temperature.

Friction on the material-tool contact surface was described by the Tresca friction model, according to which:

(2) τ=mk,

where *τ* is the shear stress on contact surface, *m* is the friction factor (set equal to *m* = 0.8 for the rolls [27] and *m* = 0.2 for other tools), *k* is the shear yield stress ($k=\sigma_{F}/\sqrt{3})$.

Thermal effects occurring during forming were considered in the analysis. The temperature of the billet was set equal to 1180 °C and the temperature of the tools was maintained constant throughout the process, at 300 °C (rolls) and at 500 °C (other tools). The coefficient of heat transfer between the tools and the material was maintained constant at 10,000 W/m^2^K.

## 3. Results

**Figure 5:**
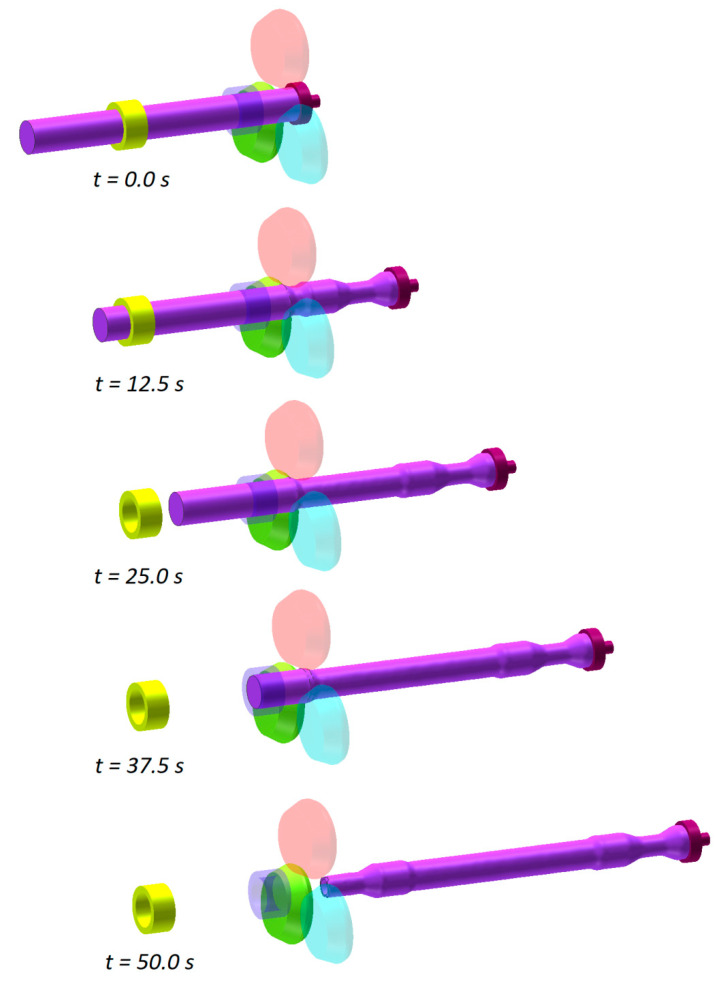
Skew rolling a solid axle in a 3-roll rolling mill.

The numerical results indicate that the rolling process runs correctly in all analyzed cases (Figure 5). The railway axle shape is correct and the forming process is free from uncontrolled slipping or necking of the formed step. Below is given a detailed description of problems that occur in the rolling of hollow and solid axles. Furthermore, the effect of the number of employed rolls on the skew-rolling process is investigated.

### 3.1. Rolling of Solid Axles

**Figure 6:**
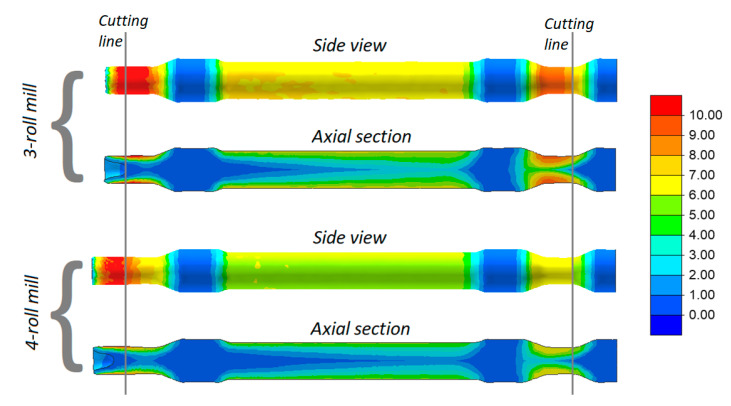
Effective strains in skew-rolled axles.

Railway axles formed in 3- and 4-roll rolling mills are shown in Figure 6. In addition, distributions of effective strains are given in the figure. An analysis of the data indicates that the strains are located in layers and their highest values occur at the surface. This strain distribution is typical of cross and skew-rolling processes, and it results from a rapid flow of the material in a circumferential direction caused by the friction forces. Higher strains occur when the solid axle is rolled with the use of 3 rolls, which results from a more considerable defect of the cross section of the workpiece. This means that the impact of the rolls must be longer in order to obtain the desired circular shape.

Figure 6 shows the location of cutting lines describing the extent of predicted allowance. The allowance located on the right side of the axle is required for chucking. The other one (located on the left of the axle) allows for the formation of an end-face cavity caused by a surface flow of the material, which is characteristic of skew-rolling processes. It can also be observed that the latter allowance is significantly bigger when rolling with 4 rolls, which indicates better elongation of the material and a more circular cross section of the workpiece than those obtained when the axles are rolled in a 3-roll rolling mill.

**Figure 7:**
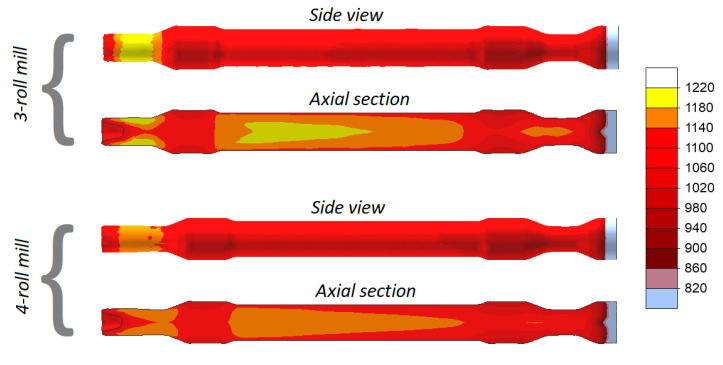
Temperatures (°C) in skew-rolled axles.

Figure 7 shows the distributions of temperature in the railway axles rolled in both 3- and 4-roll rolling mills. Despite a relatively long forming time, the temperature of the material exceeds 1100 °C (the exception being the workpiece end fixed in the chuck that is removed after rolling anyway). In the 3-roll skew-rolling process, the temperature in some regions of the workpiece is higher than the billet temperature of 1180 °C. This phenomenon is caused by the fact that large amounts of heat are generated by the work of friction and deformation. The material temperature is lower in rolling with 4 rolls, which is caused by the fact that a greater amount of heat is transferred to the rolls that—in this case—have contact with the workpiece over a bigger area.

**Figure 8:**
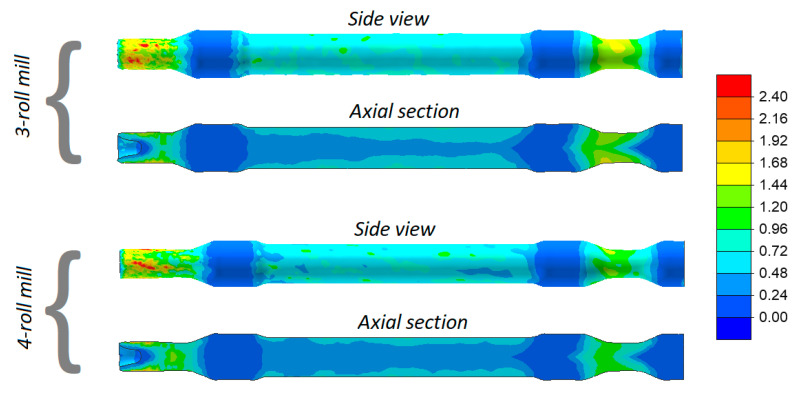
Distribution of the damage function (based on the normalized Cockcroft–Latham ductile fracture criterion) in skew rolled axles.

One of the most common failure modes in skew rolling is cracking in the axial area of the workpiece. In order to predict the probability of cracking in the analyzed cases of rolling, distributions of the damage function based on the normalized Cockcroft–Latham criterion [28] were determined (see Figure 8). It was found that the maximum values of the criterion inside the axle are smaller than 1.5. Besides the ductile fracture criterion, the critical damage is also necessary for predicting cracking. The critical damage value can be obtained by so-called calibration tests. The author’s method of rotary compression in a tool cavity [29,30] was used to determine the critical damage for the 42CrMo4 grade of steel, this value amounting to 1.777 and 2.453 for the temperature of 1100 °C and 1200 °C, respectively. A comparison of the numerical damage function and the critical damage demonstrates that the skew-rolled railway axles should be free from internal cracks.

**Figure 9:**
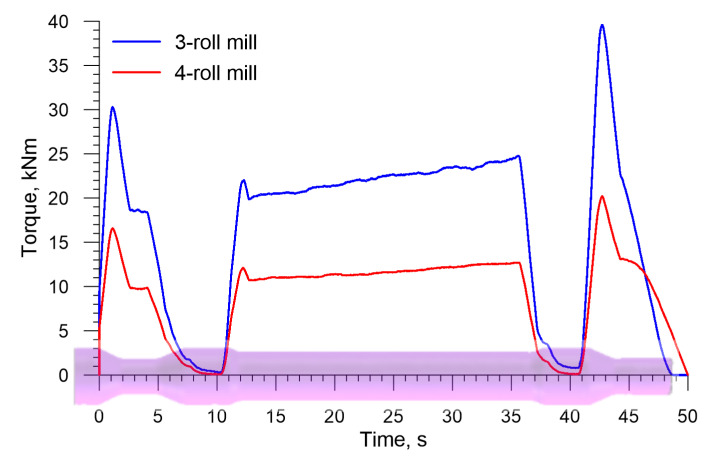
Torque on the roll in skew rolling axles in 3- and 4-roll mills.

Force parameters obtained in the simulations served as a basis for estimating energy required for producing a railway axle. Figure 9 shows the torques acting on the rolls in the analyzed rolling processes. The torque increases as the cross section of a step workpiece is reduced. The slight increase in the torque is also caused by the material cooling down, which can be observed by analyzing the torque value that is necessary to form the longest, central step on the axle. The mean torque in the 3-roll skew-rolling process is 16,547 Nm, whereas in the 4-roll skew-rolling process it is 9166 Nm. The maximum torques are respectively 39,597 Nm and 20,210 Nm. The fact that the torque is lower in the 4-roll rolling results, among others, from the use of smaller diameter tools. Based on the maximum torque, the engine power for driving the rolls can be estimated. Assuming some surplus, the power is 275 kW for a 3-roll mill and 140 kW for a 4-roll mill.

**Figure 10:**
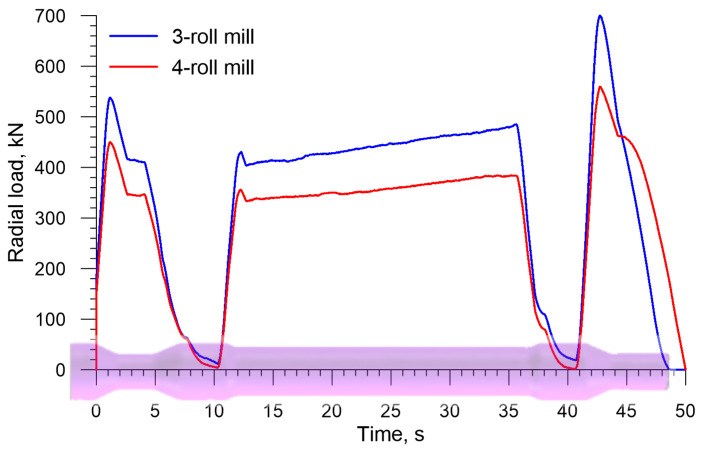
Radial load on the roll in skew rolling railway axles in 3- and 4-roll mills.

Figure 10 shows the radial loads acting on the rolls in skew rolling a solid axle; in terms of quality, they are similar to the torques. The radial load has the same direction as the vector of the roll velocity *v_R_* in Figure 2. It is worth pointing to the fact that this load is relatively low, reaching its highest values (699.7 kN and 559.4 kN for 3- and 4-roll mills, respectively) toward the end of the forming process. The radial load in the 4-roll skew-rolling process is lower by 20%, which results from a smaller material-tool contact surface due to a smaller diameter of the tool.

**Figure 11:**
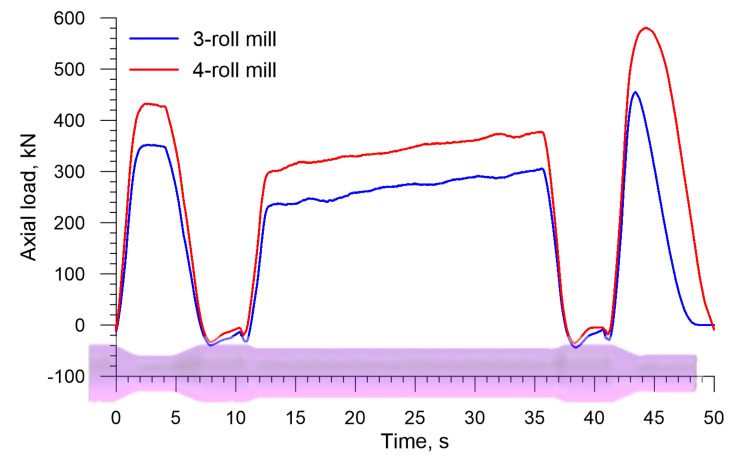
Axial load on the chuck in skew rolling railway axles in 3-and 4-roll mills.

**Figure 12:**
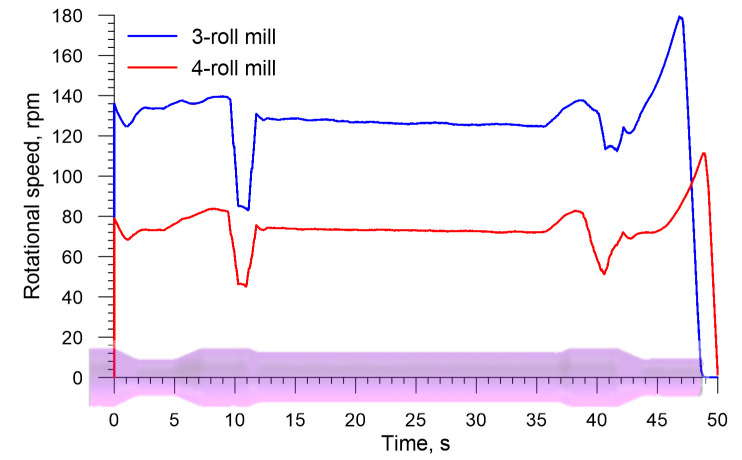
Rotational speed of the workpiece in skew rolling railway axles in 3- and 4-roll mills.

An inverse trend is observed for the axial load on the chuck (the load has the identical direction as the vector of the velocity *v_C_* in Figure 2, the distributions of which are shown in Figure 11. The drawing load in the 4-roll skew-rolling process is higher (by approximately 25%) than that in the rolling process performed with the use of 3 rolls. This phenomenon might result from the fact that the rotational speed of the workpiece is significantly lower in 4-roll skew rolling (Figure 12), which leads to a higher feed rate per one deformation cycle. The maximum axial load is 580.4 kN for a 4-roll mill and 455.1 for a 3-roll mill. An interesting fact is the occurrence of a negative load acting on the chuck when the biggest diameter steps are formed. This indicates that the rolling process runs on an independent basis and that the chuck prevents the axial movement of the workpiece.

The force parameters served as a basis for determining energy required for forming solid axles. This energy comprises the rotation work as well as the work required for radial movement of the rolls and axial movement of the chuck. These components are specified in Table 1. The results demonstrate that rolling solid axles in a 4-roll mill is more economical because it requires only 74.6% of the energy that is necessary for performing this process in a 3-roll mill.

### 3.2. Rolling of Hollow Axles

**Figure 13:**
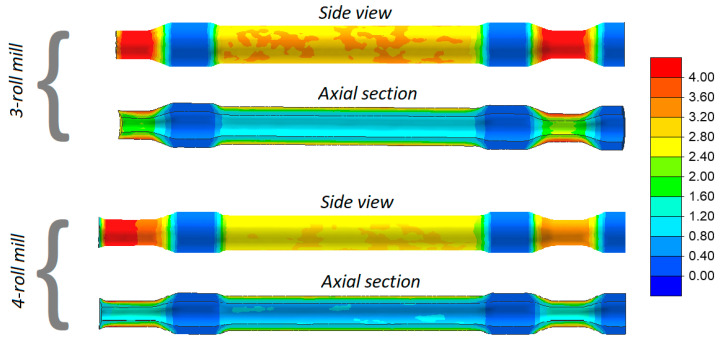
Effective strains in skew rolled hollow axles.

It can be observed that strains in the skew-rolled hollow axles are significantly lower (Figure 13). This results from the fact that a circumferential material flow does not occur because the material can easily flow in a radial direction. Nevertheless, the strains in the rolling of hollow axles (despite being 2.5 times smaller than in the rolling of solid parts) have a characteristic ring-shaped distribution. Moreover, as in the case of solid axles, the strains are slightly higher in the 3-roll skew-rolling process for hollow axles.

**Figure 14:**
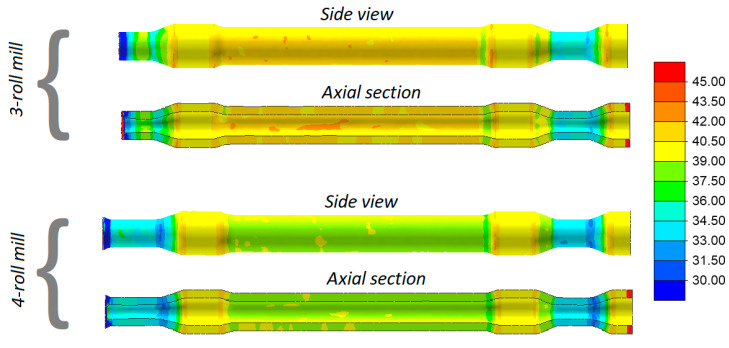
Wall thickness (given in mm) in skew-rolled hollow axles.

An analysis of axle wall thickness results given in Figure 14 provides interesting information. It can be observed that in the 3-roll rolling process the wall of the central step is slightly thicker (by approximately 1 mm), whereas in the 4-roll rolling process it undergoes a slight thinning (by approximately 1–2 mm). As for the end steps that are formed with a greater diameter reduction, the wall thinning is more considerable in both analyzed cases (3–4 mm or 4–6 mm for the 3- and the 4-roll method, respectively). As a result of the bigger necking of the walls in the 4-roll skew-rolling process, the workpiece elongation is increased and, consequently, the end step (left side of the rolled part—Figure 14) is considerably longer. This phenomenon is probably caused by a higher feed rate per one deformation cycle resulting from a lower rotational speed of the workpiece.

**Figure 15:**
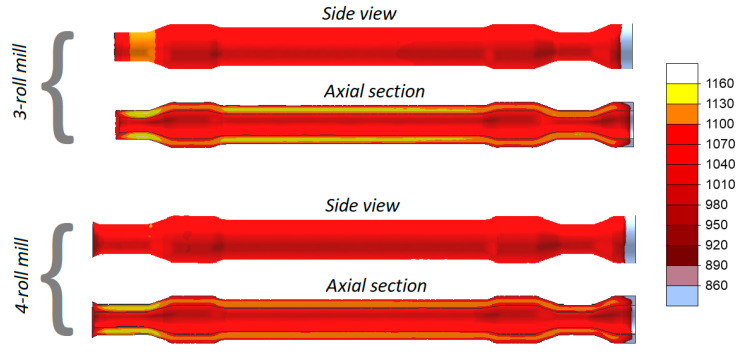
Temperature (°C) in skew-rolled hollow axles.

Despite a similar forming time (when rolling solid parts) and a considerably lower mass of the workpiece, the temperature of the workpiece does not decrease significantly. Distributions of the temperature in Figure 15 indicate that the temperature of the material in the surface layers exceeds 1070 °C, whereas inside the walls—it is higher by several °C. The trend observed for solid axles, i.e., that the axle formed with 3 rolls has a slightly higher temperature (by approximately 30 °C), can also be observed in this case.

**Figure 16:**
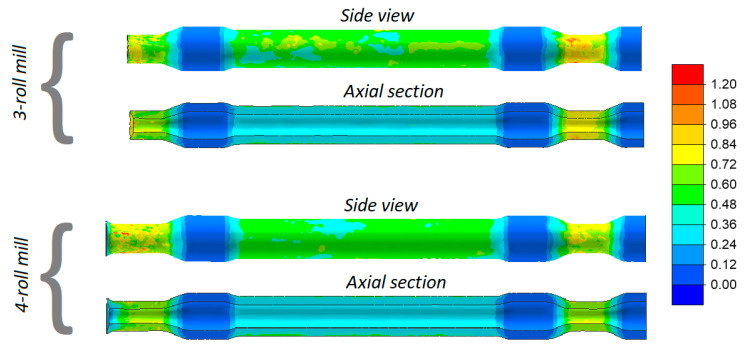
Distribution of the damage function (based on the normalized Cockcroft–Latham criterion) in skew rolled hollow axles.

The skew-rolling process for hollow axles is less susceptible to material cracking, as proved by the damage function distribution in Figure 16. The maximum values of the damage function inside the walls are up to 0.8, which means that they are more than two-fold lower than the experimental critical damage (1.777 for *T* = 1100 °C).

**Figure 17:**
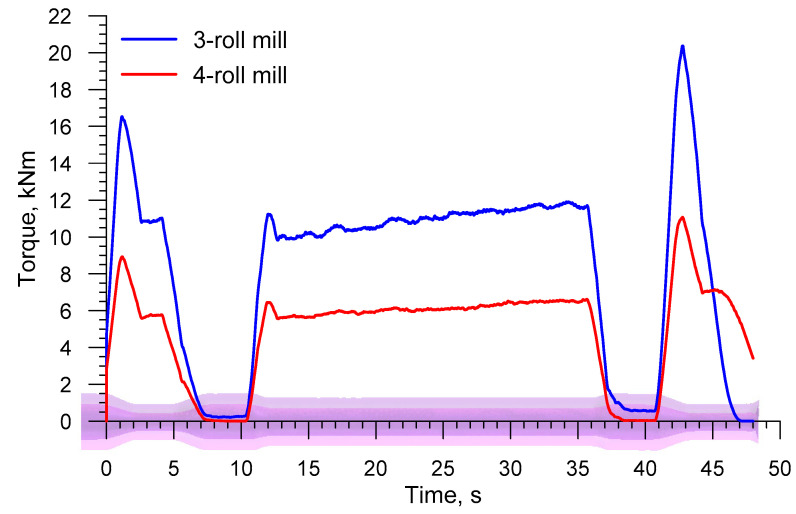
Torque on the roll in skew rolling hollow axles in 3- and 4-roll mills.

Figure 17 shows the torque on the roll in the 3- and 4-roll skew-rolling processes for hollow axles. In terms of quality, the torque is similar to that observed in the rolling of solid axles. As far as quantity is concerned, however, the torque is considerably lower. The mean torque is 8460 Nm and 4982 Nm for 3- and 4-roll rolling, respectively. This amounts, respectively, to 51.1% and 54.5% of the mean torque in the rolling of solid axles. As far as the maximum values of torque are concerned, they are: 20,377 Nm for 3-roll rolling and 11,070 Nm for 4-roll rolling.

**Figure 18:**
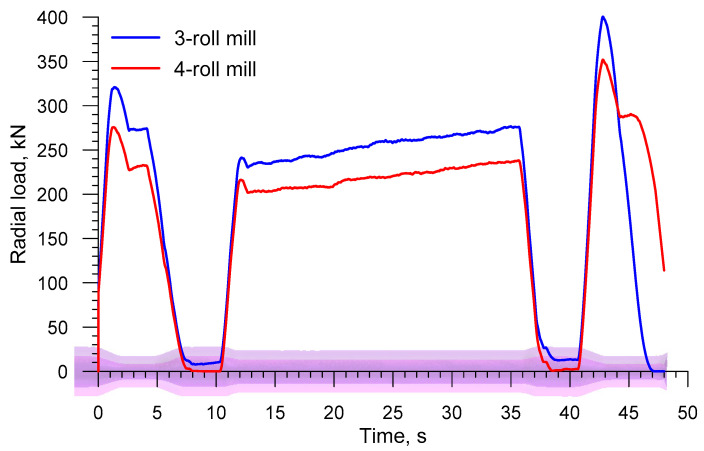
Radial load on the roll in skew rolling hollow axles in 3- and 4-roll mills.

In the skew rolling of hollow axles, radial loads moving the rolls are lower too, as shown by the plots in Figure 18. The highest radial load occurs when the end step located opposite the chuck is formed, and it is 400.4 kN and 351.9 kN for a 3- and a 4-roll mill, respectively. These values are, therefore, 40% lower than those obtained in the rolling of solid axles.

**Figure 19:**
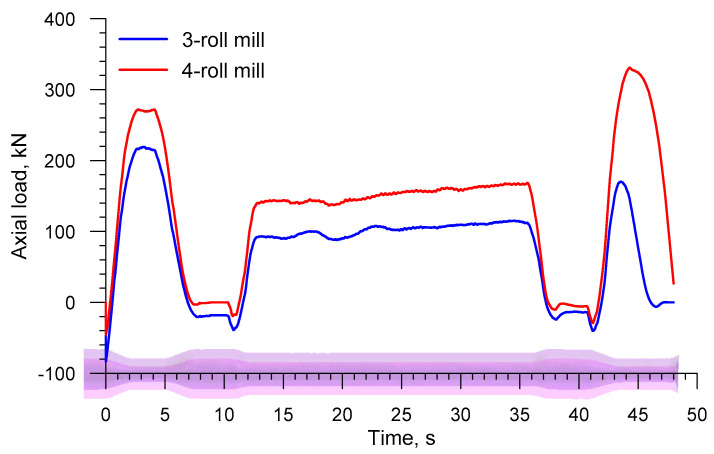
Axial load on the chuck in skew rolling hollow axles in 3- and 4-roll mills.

Figure 19 shows the axial load on the chuck in skew rolling hollow axles by 3 and 4 rolls. The behavior pattern of this load is also similar to that observed in the rolling of solid axles, including the fact that this load is negative when the biggest diameter step is formed. The highest axial load determined in the simulations is 219.3 kN for the 3-roll method and 330.7 kN for the 4-roll method.

**Figure 20:**
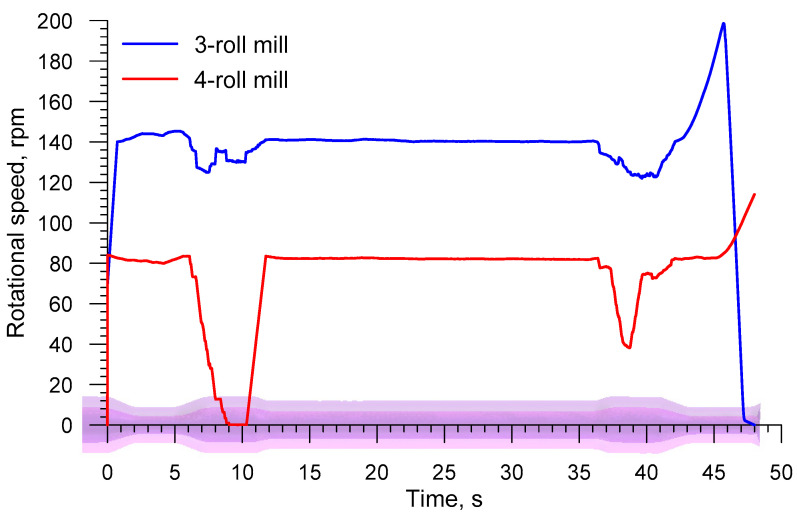
Rotational speed of the workpiece in skew rolling hollow axles in 3- and 4-roll mills.

Figure 20 shows the rotational speed of the chuck. Measured variations in this speed are similar to those in the rotational speed of the workpiece in skew rolling hollow axles. The observed decrease in this speed during the formation of the biggest steps (their diameter equal the diameter of the billet) results from reduced material-tools contact at this stage of the process. Generally speaking, the rotational speed of the workpiece deformed with 3 rolls is greater, which results from the fact that the rolls have a bigger diameter in this case.

Like in the case of solid axles, energy required for forming hollow axles was determined on the basis of obtained force parameters. Given in Table 2, the results demonstrate that the skew-rolling process for hollow axles is approximately twice less energy-consuming than that for solid axles. At the same time, the results confirm that rolling hollow axles with the use of 4 rolls is approximately 20% less energy-consuming.

When forming hollow axles, the rolling process in in a skew-rolling mill must be followed by an additional operation in which steps located in the conical region of the semi-finished product are formed. Such steps (called in this paper as “quasi-end” steps) cannot be formed on hollow axles by machining as in the case of solid axles, because it would lead to excessive thinning or even penetration of the workpiece wall. They can only be formed by rotary compression, a technique that was developed at the Lublin University of Technology and described in the publications [31,32].

**Figure 21:**
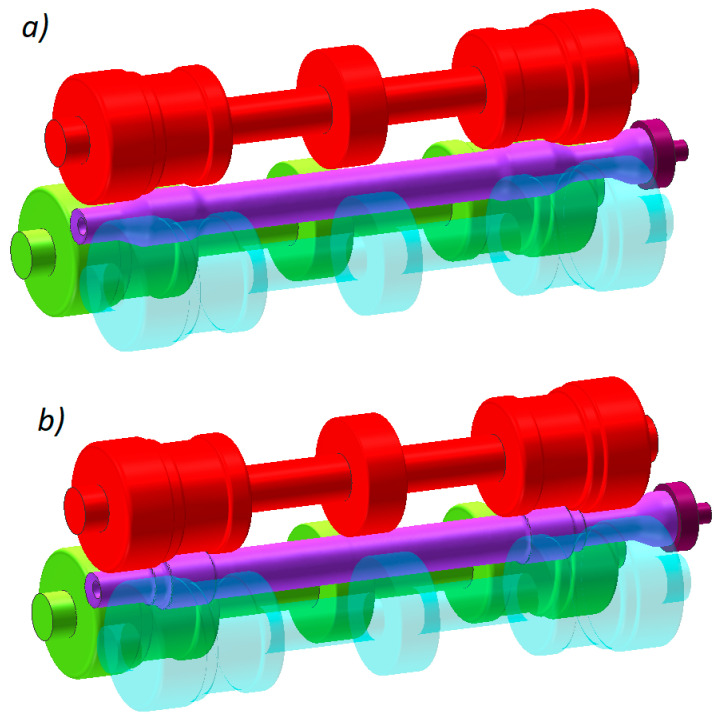
Calibration of a hollow railway axle by rotary compression: (**a**) beginning of the process, (**b**) end of the process.

Figure 21 shows the rotary compression process for a hollow railway axle. Three identical tool sets are used in this process. The tools rotate in the same direction and move toward the axis of the workpiece (here called a radial direction). Once the tools have travelled the required stroke length, their radial motion is stopped. From this moment on, the tools are only rotated to calibrate the workpiece. Each tool set consists of a shaft in which three rolls are mounted. Two of these rolls (end rolls with the maximum diameter of 500 mm) are identical and used for calibrating steps on the workpiece ends, whereas the third one (central roll) serves to straighten the axle if bent. By changing the rolls it is possible to adjust the tool set for calibrating other types of hollow axles.

The stability of the rotary compression process does not depend on the applied production method for hollow railway axles. For this reason, the numerical analysis of this process was limited to calibrating the axle produced in a 3-roll mill that, as stated above, is less accurate. Performed by Simufact.Forming, the numerical simulation of rotary compression used the geometrical model shown in Figure 21. In the analysis, it was assumed that the tool sets would move at the constant rotational speed of 60 rpm throughout the entire forming time of 3 s. Moreover, it was assumed that during the first 2 s the tools would move in a radial direction at 9 mm/s. Other parameters were identical to those applied in skew rolling.

**Figure 22:**
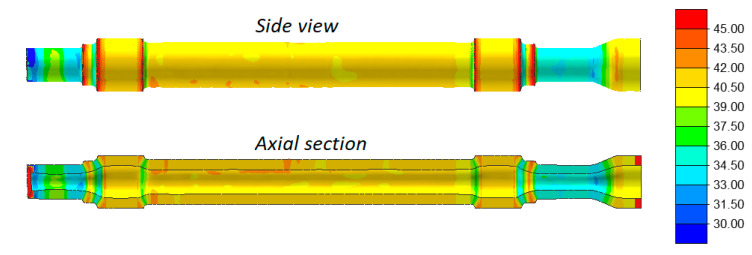
Wall thickness (given in mm) in a 3-roll skew rolled hollow axle after calibration.

The rotary-compressed axle has the required shape. All steps on this axle are calibrated correctly, as shown in Figure 22. A comparison of the hollow axles shown in Figure 13 and Figure 22 demonstrates that the end steps have undergone elongation, which results from an axial extrusion of the material from the conical region of the workpiece. An analysis of the wall thickness in the quasi-end step reveals that this wall is even several mm thicker than the wall of the end steps with the smallest diameter.

**Figure 23:**
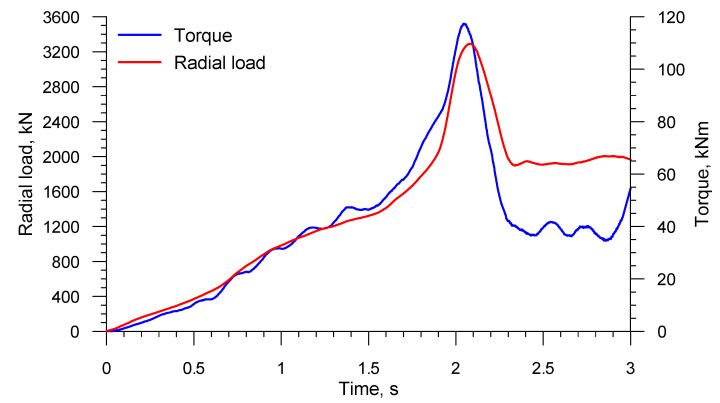
Force parameters acting on the tools during calibration of a hollow axle produced in a 3-roll mill.

Figure 23 shows the radial load and torque of the tool set used in rotary compression. It can be noticed that the force parameters gradually increase as the tools move toward the axis of the workpiece. On reaching the maximum values, which takes place when the radial motion is stopped (after 2 s), both the load and torque decrease significantly. The maximum force parameters in rotary compression are several times higher than those calculated in the rolling of axles where the workpiece–tool contact was relatively small. These values, however, are not that high to obstruct the construction of a machine for rotary compression. Furthermore, these values can be reduced either by using smaller diameter rolls or by decreasing the radial velocity of the tools.

## 4. Experimental Verification

**Figure 24:**
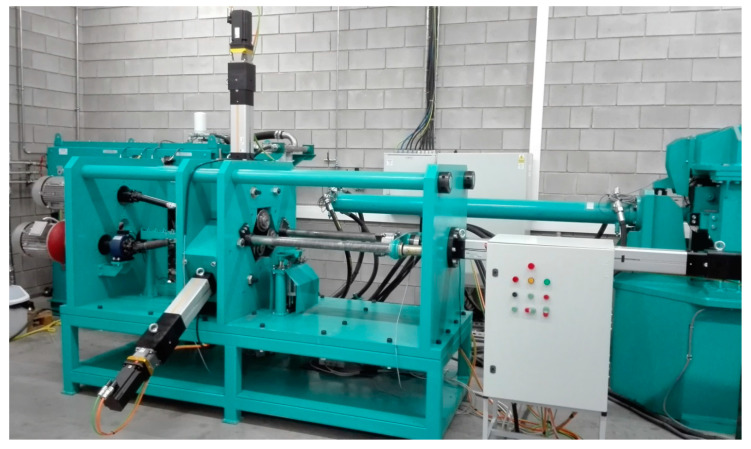
Laboratory CNC skew-rolling mill installed at the Lublin University of Technology.

The numerical results were verified experimentally, using an innovative 3-roll CNC rolling mill (Figure 24) available at the Lublin University of Technology. This machine can be used to produce axisymmetric elongated parts (shafts, axles and preforms) having up to 55 mm in diameter and 1000 mm in length. The minimum diameter that can be achieved using the standard, 150 mm diameter rolls amounts to 25 mm. The rolls are driven independently by 7.5 kW gear reducers that allow for the torque to be as high as 1200 Nm, provided that the rotational speed is maintained constant at 60 rpm. Linear motion (radial movement of the rolls or axial movement of the chuck) is ensured by electro-screw servomotors with the maximum load of 50 kN and the travel speed of up to 50 mm/s. The CNC laboratory rolling mill was equipped with a system for measuring basic force parameters in the rolling process.

**Figure 25:**
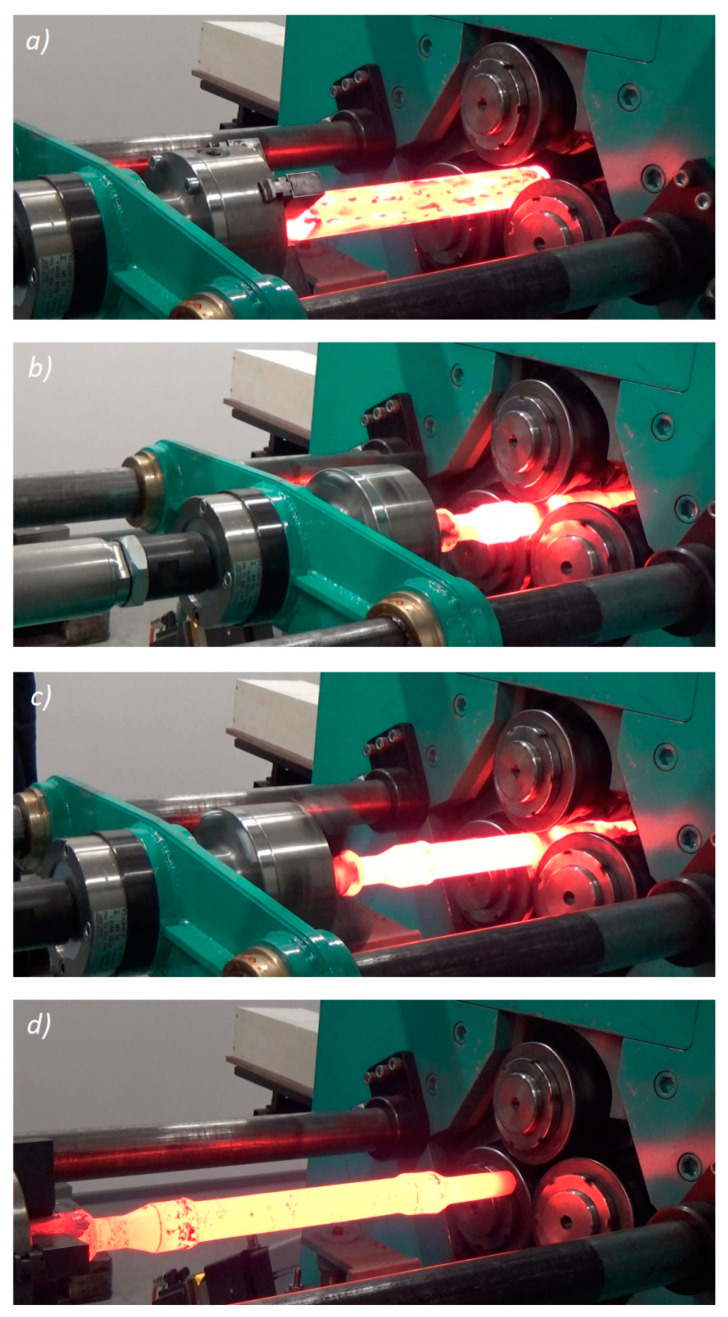
Skew rolling of a solid axle (in 1:5 scale) under laboratory conditions at the Lublin University of Technology: (**a**) billet is fed into the working space (**b**) beginning of the forming process, (**c**) end of the forming process, (**d**) rolled axle.

Given the technological capabilities of the rolling mill, the skew-rolling process was performed for a solid railway axle in a scale of 1:5. A cylindrical rod of 42 mm in diameter and 400 mm in length was used as a billet material; it was made of steel 42CrMo4 and pre-heated to 1200 °C in an electric chamber furnace. The billet was fixed in a 4-jaw chuck and fed into the workspace of the rolling mill. During rolling the chuck was moved axially with the speed of *v_x_* = 10 mm/s (when the rolls were simultaneously moving in a radial direction) or *v_x_* = 20 mm/s (when the radial position of the rolls was steady). The radial velocity *v_R_* of the tools (at *v_x_* = 10 mm/s) was selected in such a way to ensure the required product shape. One of the analyzed rolling cases is shown in Figure 25.

**Figure 26:**
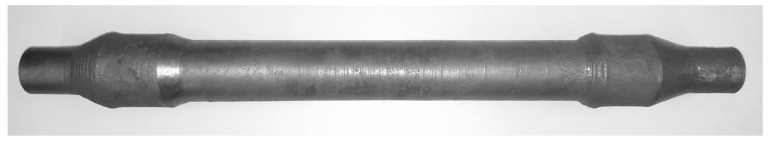
1:5 scale railway axle (after removing allowance) that was skew rolled under laboratory conditions at the Lublin University of Technology.

The axle skew rolled in a CNC rolling mill (Figure 26) has a diameter that is within a dimensional tolerance of ±0.4 mm. The axle is free from defects such as inner cracks; only some helical grooves of a small depth are visible on its outer surface (especially in the conical region where the diameter of the workpiece changed). Nevertheless, these defects are relatively easy to remove by machining.

The next step was a numerical simulation recreating the laboratory conditions of the experiments conducted at the Lublin University of Technology. To this end, the earlier described model of the real 3-roll skew rolling was modified. Selected numerical results are compared with the results of experimental tests in order to estimate the accuracy of the calculations.

**Figure 27:**
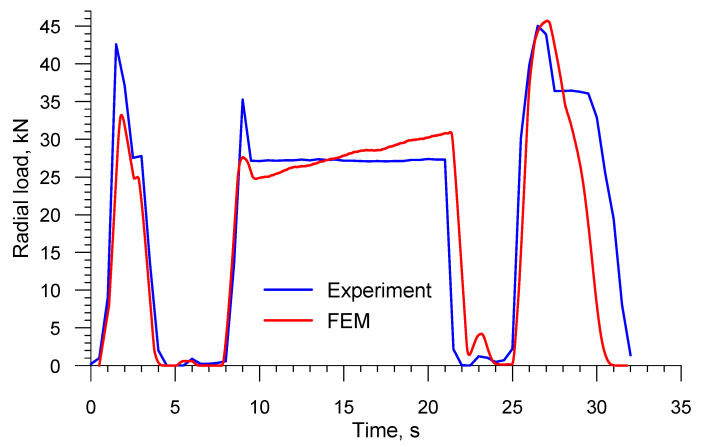
Comparison of the FEM and experimental radial loads in skew rolling railway axles in a 3-roll mill (1:5 scale).

Figure 27 presents the numerical and experimental radial loads acting on the roll. A comparison of these values shows a very good qualitative agreement. The highest radial load (approximately 46 kN) is measured toward the end of the process when the end step opposite the chuck is formed. In order to perform a quantitative assessment of the results, the mean radial load is determined, its value being 20.02 kN and 19.75 kN in the experiment and the numerical analysis, respectively. These values are nearly in total agreement (the difference between the loads is only 1.3%).

**Figure 28:**
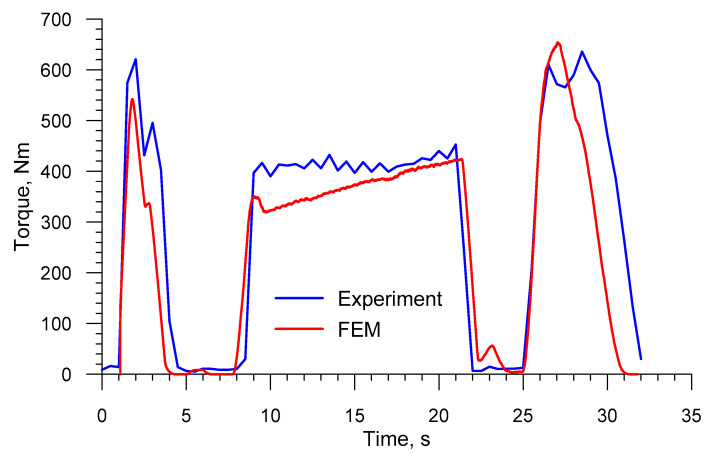
Comparison of the FEM and experimental torques in skew rolling railway axles in a 3-roll mill (1:5 scale).

Figure 28 shows the comparison of the FEM and experimental torques that are qualitatively very similar to the FEM and experimental radial loads. The mean experimental torque is 301.19 Nm and is higher than the mean torque from the numerical analysis, which amounted to 270.74 Nm. The values differ by 10.1%. Finally, considering the agreement between the experimental and numerical radial loads and torques, it can be claimed that the developed numerical model of a skew-rolling process for a railway axle properly reflects the real forming conditions.

## 5. Conclusions

The results of numerical calculations and experimental testing lead to the following conclusions:

- both hollow and solid railway axles can be successfully skew rolled in both 3- and 4-roll rolling mills;
- skew rolling in a CNC rolling mill is highly effective and universal, and the forming forces and torques in this process are relatively low;
- Skew-rolled hollow axles must be re-calibrated in order to form end steps;
- The axles formed in a 4-roll rolling mill undergo a more significant elongation than those produced in a machine with 3 tools; this results in a smaller defect of the cross section (solid axles) or a lower thickness of the wall (hollow axles);
- Skew rolling in a 4-roll mill is less energy-consuming (by approximately 20–25%), with lower loads on the roll (by approximately 20–40%) and higher loads on the chuck (by approximately 25–50%);
- Experimental verification of the numerical model of the analyzed skew-rolling process (performed under laboratory conditions at the Lublin University of Technology) has confirmed the correctness of the numerical results.

## Figures and Tables

**Table 1:** Energy required to form solid axles.

Type of Rolling Mill	Energy Component, kJ			Total Energy, kJ
	Rotational Motion of the Rolls	Radial Motion of the Rolls	Axial Motion of the Chuck	
3-roll	3 × 5195.9	3 × 52.8	96.2	15,842.3
4-roll	4 × 2878.2	4 × 43.7	134.8	11,822.4

**Table 2:** Energy required to form hollow axles.

Type of Rolling Mill	Energy Component, kJ			Total Energy, kJ
	Rotational Motion of the Rolls	Radial Motion of the Rolls	Axial Motion of the Chuck	
3-roll	3 × 2550.2	3 × 30.1	37.9	7778.8
4-roll	4 × 1504.8	4 × 26.8	66.3	6192.7
